# Supplementary material for: Concentration- and Time-Dependent Dietary Exposure to Graphene Oxide and Silver Nanoparticles: Effects on Food Consumption and Assimilation, Digestive Enzyme Activities, and Body Mass in Acheta domesticus
Source: Insects. 2024 Jan 29;15(2):89. doi: 10.3390/insects15020089 (PMC10888715; doi:10.3390/insects15020089)
Supplement: Supplementary file 1 [file insects-15-00089-s001.zip › insects-2814266-supplementary.pdf]

insects MDPI

Supplementary Materials

Concentration- and Time-Dependent Dietary Exposure to Graphene Oxide and Silver Nanoparticles: Effects on Food Consumption and Assimilation, Digestive Enzyme Activities, and Body Mass in *Acheta domesticus*

Reyhaneh Seyed Alian [1], Barbara Flasz [1], Andrzej Kędziorski [1], Łukasz Majchrzycki [2] and Maria Augustyniak [1,*]

[1] Institute of Biology, Biotechnology and Environmental Protection, University of Silesia in Katowice, 40-007 Katowice, Poland
[2] Institute of Physics, Faculty of Materials Engineering and Technical Physics, Poznan University of Technology, Piotrowo 3, 60-965 Poznan, Poland
* Correspondence: maria.augustyniak@us.edu.pl

COMPOSITION OF RABBIT FEED

KDT (for growing rabbits) by UNIPASZ Company (Poland) is used routinely for rearing a continuous laboratory colony of cricket, *Acheta domesticus* L.

The feed is rich in easily assimilated mineral nutrients, free of GMO and contains c.a. 55 % carbohydrates of plant origin.

Analytical composition (per 1 kg):

Raw compounds: proteins - 17 %, fibre – 14.5 %, fat – 3.50 %, ash – 7.50 %

Minerals: Ca – 0.95 %, Na – 0.19 %, P – 0.50 %, Zn – 85 mg, Fe – 25 mg, other – c.a. 100 mg

Some additives

Vitamins: A – 12,000 mass units, E – 60 mg, B (complex) – c.a. 60 mg, Biotin – 200 mcg, C – 250 mg;

Aminoacids: Methionine – 750 mg, L-Lysine – 3700 mg, L-Threonine – 1600 mg

Antioxidants: 2.95 mg

Table S1. Permutational analysis of variance (PERMANOVA) test considering the main factors: 'NP type' 'Concentration' and 'Time', and their interaction, influencing Food consumption and Food assimilation measured in *Acheta domesticus* following exposure to GO or AgNPs in the diet *(PERMANOVA was performed with 999 permutations, using the adonis2 function in R vegan package; F – F ratio, df – degrees of freedom, p – p-value, n = 5).*

**Effect**	**Food consumption**			**Food assimilation**		
	**F**	***df***	**p**	**F**	***df***	**p**
NPs type [1]	13.239	1	0.001	13.795	1	0.001
Concentration [2]	5.444	3	0.001	3.654	3	0.002
Time [3]	43.003	3	0.001	27.066	3	0.001
[1] × [2]	5.506	6	0.001	7.262	6	0.001
[1] × [3]	3.749	6	0.001	2.986	6	0.001
[2] × [3]	1.529	18	0.061	1.830	18	0.003
[1] × [2] × [3]	1.967	18	0.006	1.891	18	0.003
Residual		270			270	
Total		325			325	

Table S2. Food consumption - Results of the Tukey post-hoc test performed after multivariate repeated measures Analysis of Variance (Homogeneous groups are marked with gray boxes).

Multivariate repeated measures Analysis of Variance – Tukey test (Food consumption)

NPs	Conc.	Time	Mean
Ag	C3	(16-21)	10.865
Ag	C2	(11-16)	11.175
Ag	C3	(11-16)	12.245
Ag	C2	(9-11)	12.874
GO	C1	(11-16)	14.994
Ag	C2	(16-21)	15.065
GO	C2	(5-7)	15.489
Ag	C1	(11-16)	16.112
Ag	C1	(16-21)	16.984
Ag	C3	(9-11)	17.485
GO	C3	(5-7)	18.486
Ag	C2	(7-9)	19.082
GO	C1	(16-21)	19.128
GO	C2	(16-21)	20.442
Ag	C0	(16-21)	20.978
GO	C0	(16-21)	20.978
GO	C2	(11-16)	21.373
GO	C0	(9-11)	21.650
Ag	C0	(9-11)	21.650
GO	C0	(11-16)	22.027
Ag	C0	(11-16)	22.027
GO	C3	(11-16)	22.392
Ag	C3	(7-9)	22.405
Ag	C2	(5-7)	22.531
Ag	C2	(3-5)	22.781
GO	C3	(9-11)	22.892
Ag	C1	(9-11)	22.934
GO	C1	(9-11)	23.277
GO	C3	(16-21)	23.578
GO	C2	(7-9)	24.418
Ag	C0	(7-9)	24.701
GO	C0	(7-9)	24.701
Ag	C0	(5-7)	25.717
GO	C0	(5-7)	25.717
GO	C1	(5-7)	26.399
Ag	C2	(1-3)	26.868
GO	C2	(9-11)	27.113
Ag	C1	(7-9)	27.427
GO	C2	(3-5)	28.731
GO	C1	(7-9)	28.991
GO	C3	(7-9)	31.449
Ag	C1	(5-7)	33.171
Ag	C3	(5-7)	33.770
GO	C1	(3-5)	34.536
GO	C2	(1-3)	35.969
Ag	C0	(1-3)	36.979
GO	C0	(1-3)	36.979
GO	C3	(3-5)	39.068
Ag	C3	(1-3)	39.355
Ag	C3	(3-5)	41.333
GO	C1	(1-3)	41.743
GO	C0	(3-5)	42.020
Ag	C0	(3-5)	42.020
Ag	C1	(1-3)	42.470
Ag	C1	(3-5)	42.908
GO	C3	(1-3)	43.077

Homogeneity

Table S3. Food assimilation - Results of the Tukey post-hoc test performed after multivariate repeated measures Analysis of Variance (Homogeneous groups are marked with gray boxes).

Multivariate repeated measures Analysis of Variance – Tukey test (Food assimilation)

NPs	Ag	Ag	Ag	Ag	Ag	GO	Ag	GO	Ag	Ag	GO	Ag	GO	Ag	Ag	Ag	GO	GO	Ag	Ag	GO	Ag	GO	Ag	GO	GO	GO	GO	GO	Ag	GO	Ag	Ag	GO	GO	GO	GO	GO	Ag	GO	GO	GO	Ag	GO	Ag	GO	Ag	Ag	Ag	GO	GO	Ag	Ag	Ag	GO	GO
Conc.	C2	C3	C3	C2	C2	C0	C0	C1	C3	C1	C0	C0	C2	C1	C2	C2	C3	C1	C3	C0	C0	C2	C1	C0	C0	C2	C2	C3	C2	C0	C0	C1	C2	C3	C1	C3	C2	C1	C1	C2	C3	C1	C0	C0	C0	C0	C3	C3	C1	C2	C3	C3	C1	C1	C3	C1
Time	(11-16)	(16-21)	(11-16)	(16-21)	(9-11)	(16-21)	(16-21)	(16-21)	(9-11)	(11-16)	(9-11)	(9-11)	(5-7)	(16-21)	(5-7)	(3-5)	(5-7)	(11-16)	(7-9)	(11-16)	(11-16)	(7-9)	(9-11)	(5-7)	(5-7)	(16-21)	(11-16)	(11-16)	(7-9)	(7-9)	(7-9)	(9-11)	(1-3)	(9-11)	(5-7)	(16-21)	(3-5)	(3-5)	(7-9)	(9-11)	(7-9)	(7-9)	(1-3)	(1-3)	(3-5)	(3-5)	(1-3)	(5-7)	(5-7)	(1-3)	(3-5)	(3-5)	(3-5)	(1-3)	(1-3)	(1-3)
Mean	3.557	5.850	6.094	6.774	7.082	7.408	7.408	8.056	8.173	8.537	8.566	8.566	8.642	9.020	9.196	9.393	9.457	9.469	9.768	10.307	10.307	10.870	10.885	11.052	11.052	11.169	11.646	11.911	12.204	12.696	12.696	12.802	13.088	13.353	13.513	13.831	14.586	15.674	15.931	16.576	17.044	17.493	17.557	17.557	17.911	17.911	18.469	18.703	19.294	19.368	20.005	20.355	20.629	21.011	22.339	22.728
Homogeneity	■	■	■	■	■	■	■	■	■	■	■	■	■	■	■	■	■	■	■	■	■	■	■	■	■	■	■	■	■	■	■	■	■	■	■	■	■																			
		■	■	■	■	■	■	■	■	■	■	■	■	■	■	■	■	■	■	■	■	■	■	■	■	■	■	■	■	■	■	■	■	■	■	■	■	■	■	■	■	■														
		■	■	■	■			■	■	■	■	■	■	■	■	■	■	■	■	■	■	■	■	■	■	■	■	■	■	■	■	■	■	■	■	■	■	■	■	■	■	■	■	■	■												
			■	■	■			■	■	■	■	■	■	■	■	■	■	■	■	■	■	■	■	■	■	■	■	■	■	■	■	■	■	■	■	■	■	■	■	■	■	■	■	■	■	■	■										
				■	■	■	■	■	■	■	■	■	■	■	■	■	■	■	■	■	■	■	■	■	■	■	■	■	■	■	■	■	■	■	■	■	■	■	■	■	■					■	■								
									■				■		■	■		■	■	■	■	■	■	■	■	■	■	■	■	■	■	■	■	■	■	■	■	■	■	■	■	■	■	■	■	■	■	■	■	■					
						■	■	■	■	■	■	■	■	■	■	■	■	■	■	■	■	■	■	■	■	■	■	■	■	■	■	■	■	■	■	■	■	■	■	■	■	■					■	■		■						
										■	■	■	■	■	■	■	■	■	■	■	■	■	■	■	■	■	■	■	■	■	■	■	■	■	■	■	■	■	■	■	■	■	■	■	■	■	■	■		■		■				
													■		■	■			■			■	■	■	■	■	■	■	■	■	■	■	■	■	■	■	■	■	■	■	■	■	■	■	■	■	■	■	■	■	■	■			
															■	■			■			■	■			■	■	■	■	■	■	■	■	■	■	■	■	■	■	■	■	■	■	■	■	■	■	■	■	■	■	■	■	■		
																						■				■	■		■	■	■	■	■	■	■	■	■	■	■	■	■	■	■	■	■	■	■	■	■	■	■	■	■	■	■	■

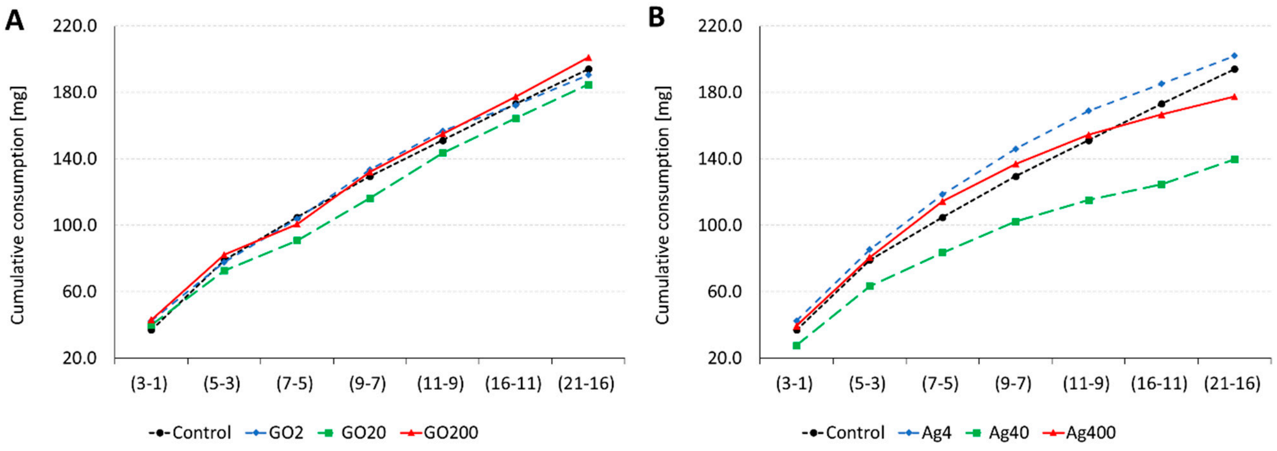

Figure S1. Cumulative food consumption (mg dry weight per individual; mean) in *A. domesticus* treated with nanoparticles: A) graphene oxide (GO) and B) silver nanoparticles (AgNPs) measured in intervals.

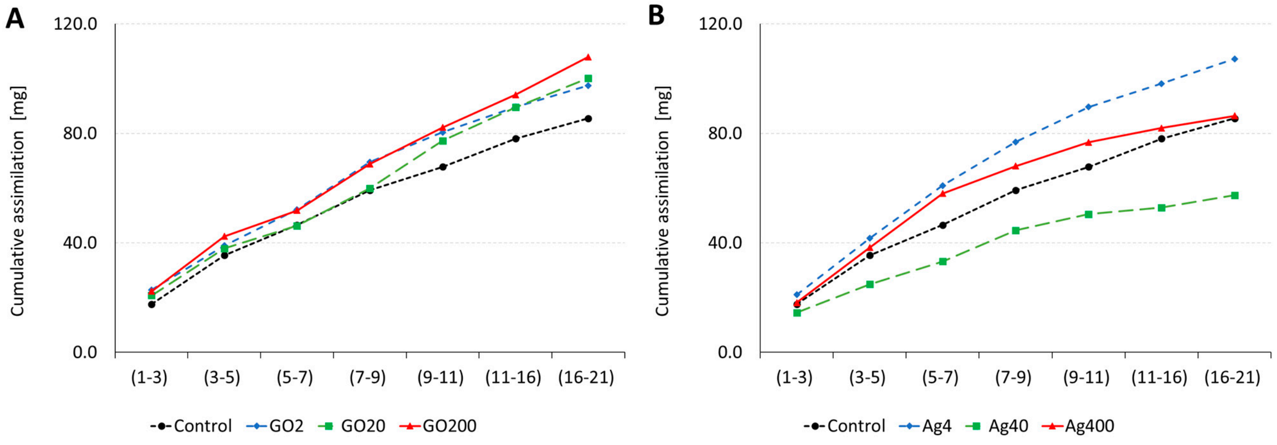

Figure S2. Cumulative food assimilation (mg dry weight per individual; mean) in *A. domesticus* treated with nanoparticles: A) graphene oxide (GO) and B) silver nanoparticles (AgNPs) measured in intervals.

Table S4A. Multivariate repeated measures Analysis of Variance for 'NPstype', 'Concentration', and interaction of both factors, on digestive enzymes activity in *A. domesticus*, measured at 1-5, 16 and 21 day of exposure (*F – F ratio, df_1 and df_2 – treatment and error degrees of freedom, respectively, p – p value, n = 5*).

Effect	Amylase				Lipase				Protease			
	F	df_1	df_2	p	F	df_1	df_2	p	F	df_1	df_2	p
NPs type [1]	46.113	1	30	<0.001	164.369	1	25	<0.001	23.42	1	30	<0.001
Concentration [2]	427.046	3	30	<0.001	145.687	3	25	<0.001	28.33	3	30	<0.001
[1] × [2]	12.437	3	30	<0.001	27.797	3	25	<0.001	4.72	3	30	0.008
Time [3]	3.544	6	25	0.002	15.253	6	20	<0.001	69.51	6	25	<0.001
[3] × [1]	3.823	6	25	0.001	14.171	6	20	<0.001	6.98	6	25	<0.001
[3] × [2]	2.203	18	71	0.005	4.149	18	57	<0.001	3.84	18	71	<0.001
[3] × [1] × [2]	2.726	18	71	<0.001	7.542	18	57	<0.001	5.68	18	71	<0.001

Table S4B. Continuation of Table S4A.

Effect	α-Glu				β-Glu				β-Gal			
	F	df_1	df_2	p	F	df_1	df_2	p	F	df_1	df_2	p
NPs type [1]	23.554	1	32	<0.001	63.992	1	31	<0.001	33.647	1	31	<0.001
Concentration [2]	26.301	3	32	<0.001	11.668	3	31	<0.001	14.737	3	31	<0.001
[1] × [2]	3.995	3	32	0.016	46.522	3	31	<0.001	31.149	3	31	<0.001
Time [3]	23.378	6	27	<0.001	3.439	6	26	0.003	11.191	6	26	<0.001
[3] × [1]	6.896	6	27	<0.001	4.345	6	26	<0.001	4.397	6	26	<0.001
[3] × [2]	2.318	18	77	0.003	3.244	18	74	<0.001	3.856	18	74	<0.001
[3] × [1] × [2]	3.854	18	77	<0.001	2.630	18	74	<0.001	2.466	18	74	0.001

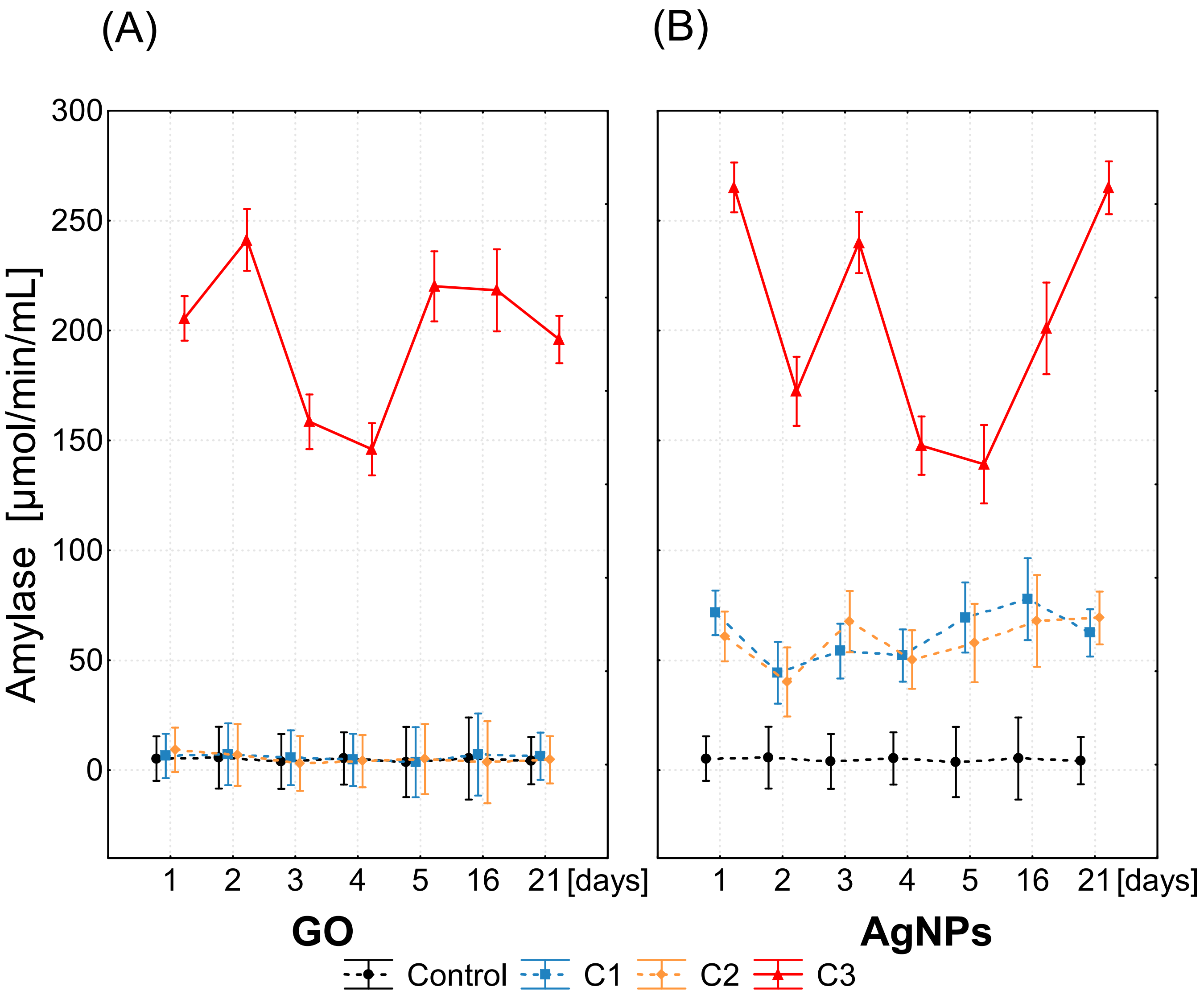

Figure S3. α-amylase activity (mean ± SE) in samples from the digestive tract of *A. domesticus* from control and nanoparticles-treated groups. A) graphene oxide (GO) and B) silver nanoparticles (AgNPs).

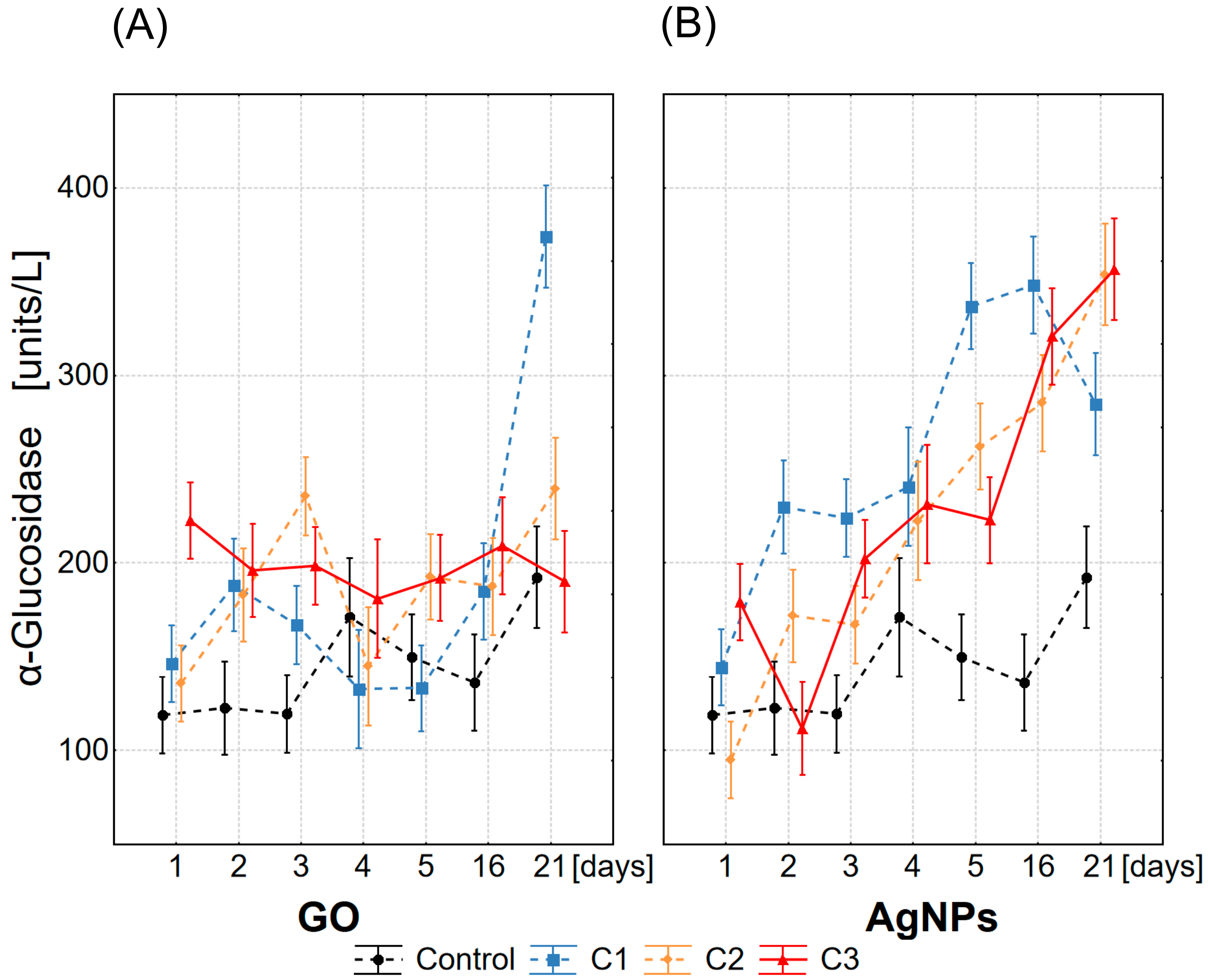

Figure S4. α-glucosidase activity (mean ± SE) in samples from the digestive tract of *A. domesticus* from control and nanoparticles-treated groups. A) graphene oxide (GO) and B) silver nano-particles (AgNPs).

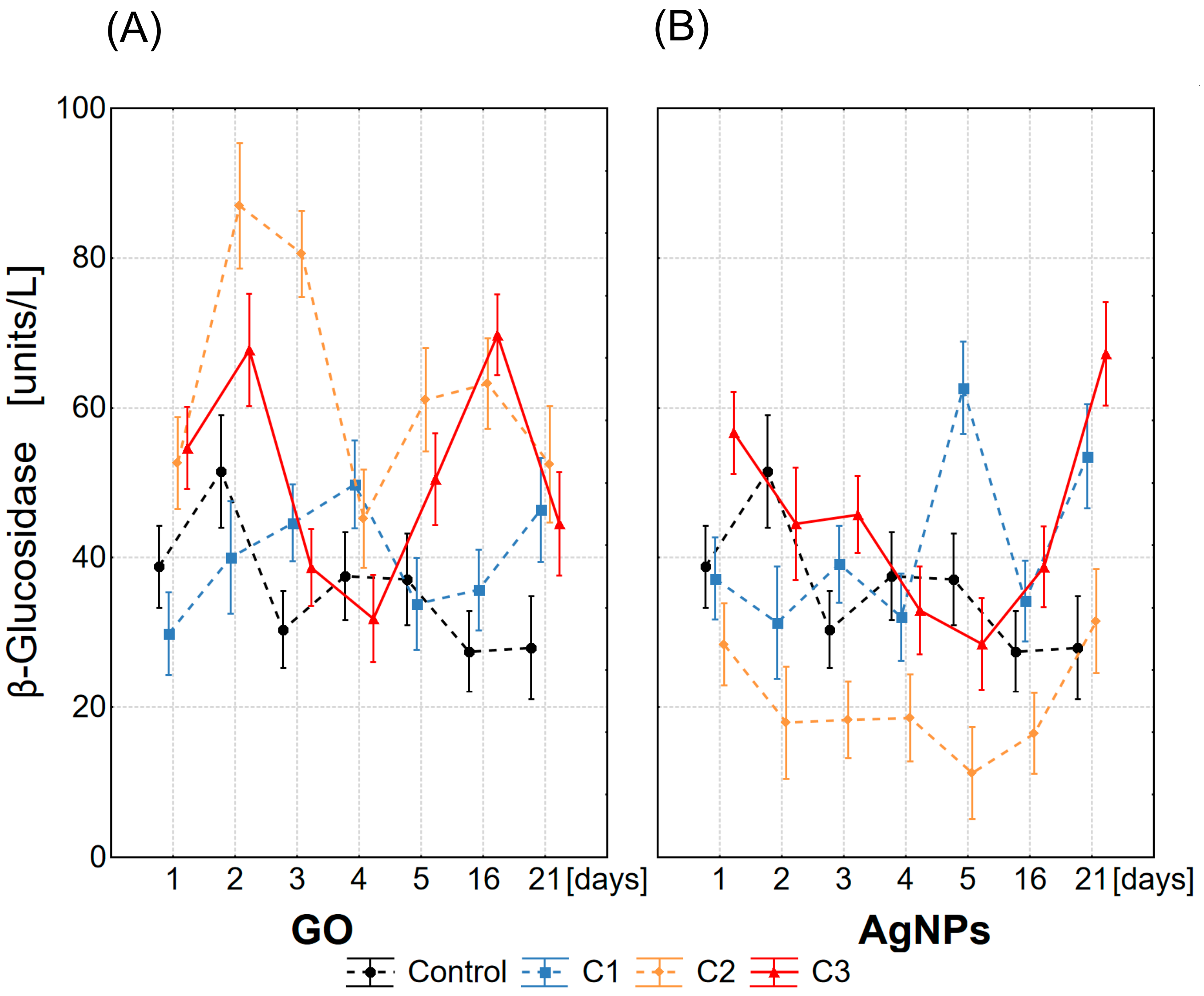

Figure S5. β-glucosidase activity (mean ± SE) in samples from the digestive tract of *A. domesticus* from control and nanoparticles-treated groups. A) graphene oxide (GO) and B) silver nano-particles (AgNPs).

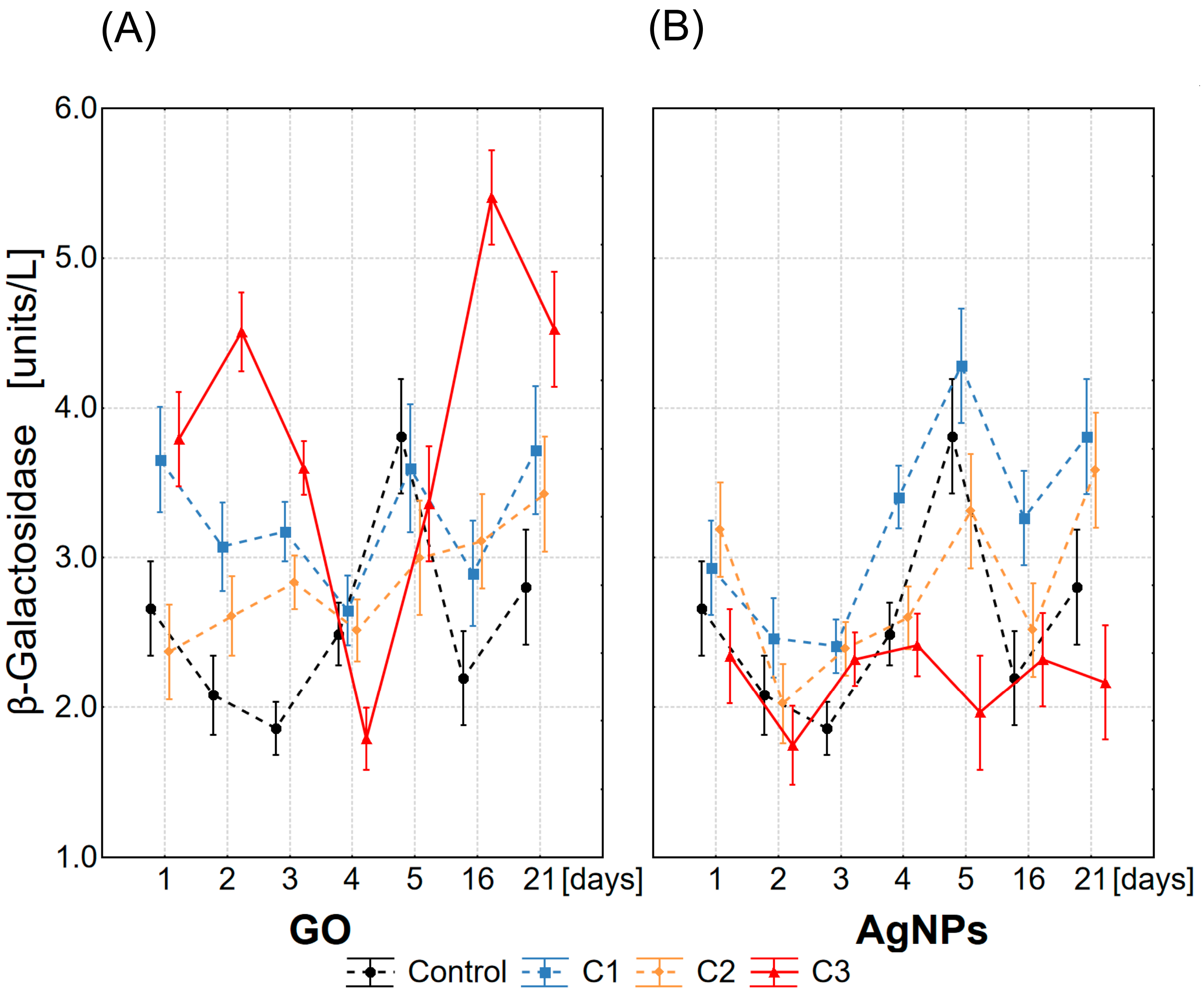

Figure S6. β-galactosidase activity (mean ± SE) in samples from the digestive tract of *A. domesticus* from control and nanoparticles-treated groups. A) graphene oxide (GO) and B) silver nano-particles (AgNPs).

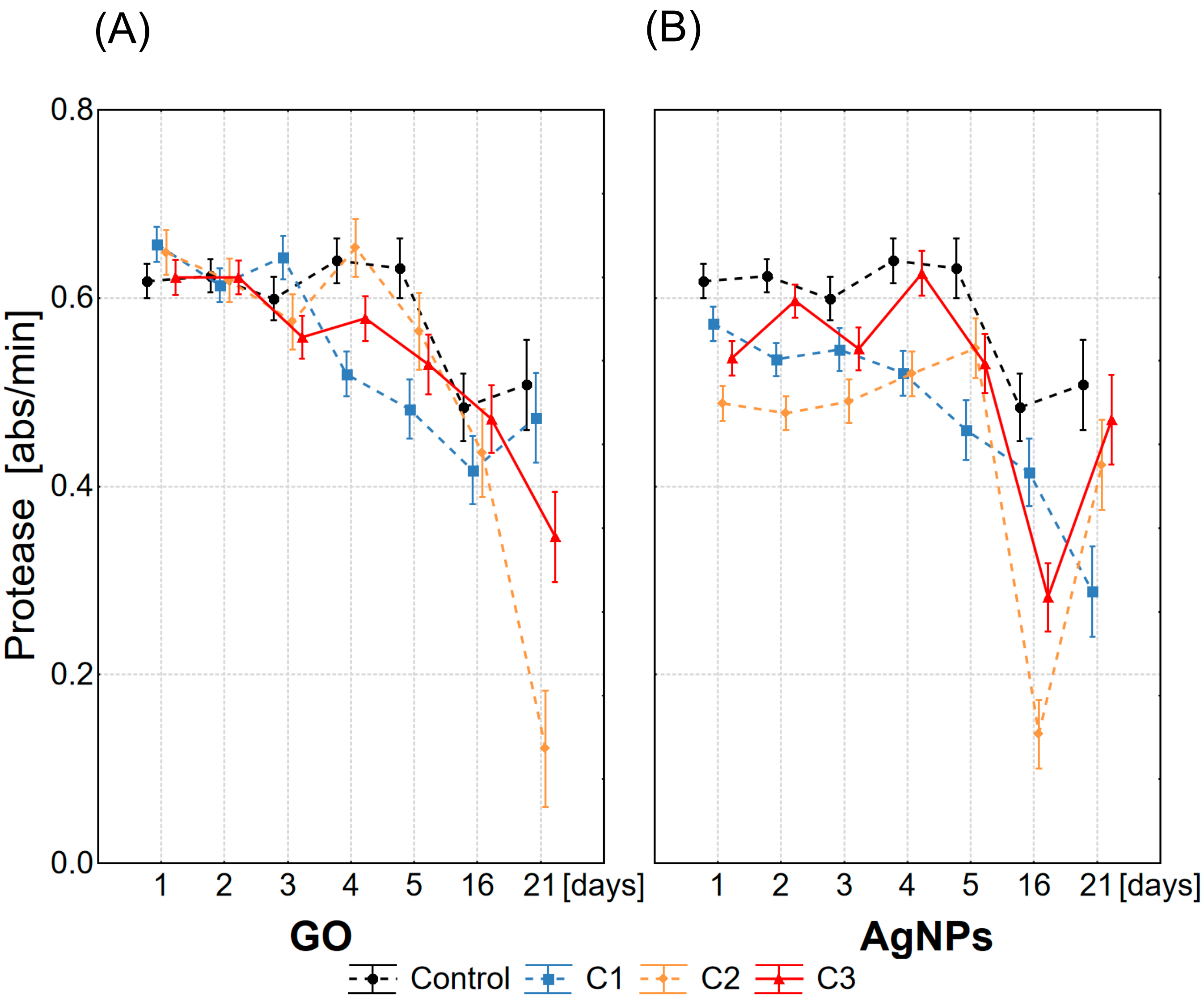

Figure S7. Protease activity (mean ± SE) in samples from the digestive tract of *A. domesticus* from control and nanoparticles-treated groups. A) graphene oxide (GO) and B) silver nano-particles (AgNPs).

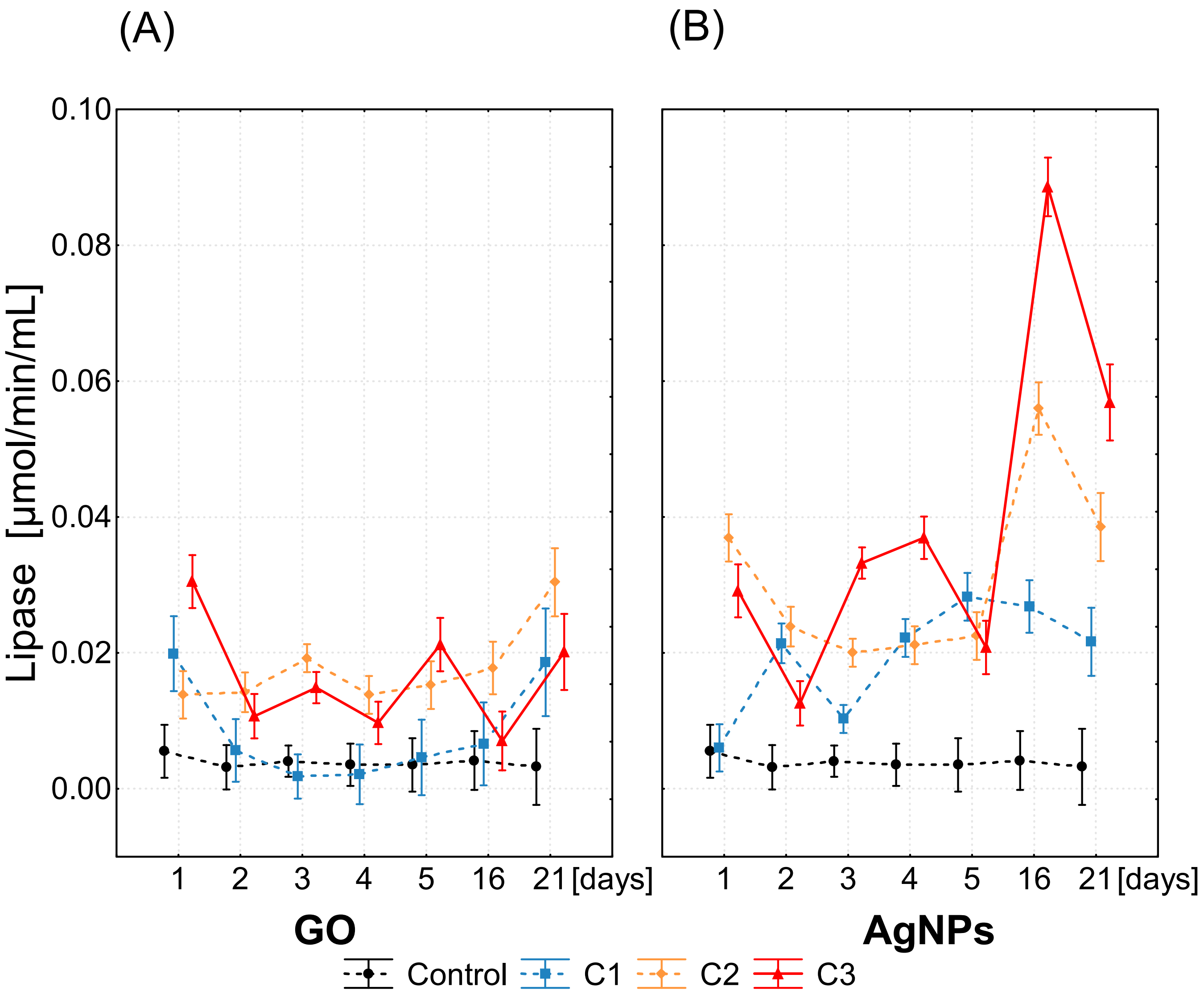

Figure S8. Lipase activity (mean ± SE) in samples from the digestive tract of *A. domesticus* from control and nanoparticles-treated groups. A) graphene oxide (GO) and B) silver nanoparticles (AgNPs).

Table S5. Amylase - Results of the Tukey post-hoc test performed after multivariate repeated measures Analysis of Variance (Homogeneous groups are marked with gray boxes).

Multivariate repeated measures Analysis of Variance – Tukey test (Amylase)

NPs	GO	GO	GO	GO	Ag	Ag	GO	GO	GO	Ag	GO	GO	GO	Ag
Conc.	C2	C2	C1	C0	C0	C0	C0	C2	C0	C0	C1	C2	C2	C0
Time	t3	t16	t5	t5	t5	t3	t3	t4	t21	t21	t4	t21	t5	t1
Mean	3.02	3.60	3.61	3.69	3.69	3.96	3.96	4.09	4.31	4.31	4.65	4.70	5.00	5.23

NPs	GO	GO	Ag	Ag	GO	GO	Ag	GO	GO	GO	GO	GO	GO	GO
Conc.	C0	C0	C0	C0	C0	C1	C0	C0	C1	C1	C2	C1	C1	C2
Time	t1	t16	t16	t4	t4	t3	t2	t2	t21	t1	t2	t16	t2	t1
Mean	5.23	5.29	5.29	5.32	5.32	5.63	5.69	5.69	6.30	6.46	6.91	7.08	7.22	9.25

NPs	Ag	Ag	Ag	Ag	Ag	Ag	Ag	Ag	Ag	Ag	Ag	Ag	Ag	Ag
Conc.	C2	C1	C2	C1	C1	C2	C2	C1	C2	C2	C2	C1	C1	C1
Time	t2	t2	t4	t4	t3	t5	t1	t21	t3	t16	t21	t5	t1	t16
Mean	40.12	44.30	50.32	52.14	54.15	57.81	60.81	62.44	67.57	67.89	69.22	69.42	71.55	77.79

NPs	Ag	GO	Ag	GO	Ag	GO	Ag	GO	GO	GO	Ag	GO	Ag	Ag
Conc.	C3	C3	C3	C3	C3	C3	C3	C3	C3	C3	C3	C3	C3	C3
Time	t5	t4	t4	t3	t2	t21	t16	t1	t16	t5	t3	t2	t21	t1
Mean	139.17	145.99	147.63	158.47	172.33	195.91	200.98	205.50	218.31	220.10	240.04	241.20	264.93	265.09

Homogeneity

Table S6. α-Glucosidase - Results of the Tukey post-hoc test performed after multivariate repeated measures Analysis of Variance (Homogeneous groups are marked with gray boxes).

Multivariate repeated measures Analysis of Variance – Tukey test (α-Glucosidase)

NPs	Ag	Ag	GO	Ag	GO	Ag	GO	Ag	GO	GO	GO	Ag	GO	Ag
Conc.	C2	C3	C0	C0	C0	C0	C0	C0	C1	C1	C2	C0	C0	C1
Time	t1	t2	t1	t1	t3	t3	t2	t2	t4	t5	t1	t16	t16	t1
Mean	94.77	111.57	118.66	118.66	119.41	119.41	122.45	122.45	132.66	133.07	135.68	136.11	136.11	144.14
Homogeneity	■	■	■	■	■	■	■	■	■	■	■	■	■	■
		■	■	■	■	■	■	■	■	■	■	■	■	■
					■	■	■	■	■	■	■	■	■	■
														■

NPs	GO	GO	GO	Ag	GO	Ag	GO	Ag	Ag	Ag	GO	GO	GO	GO
Conc.	C2	C1	C0	C0	C1	C2	C0	C0	C2	C3	C3	C2	C1	C2
Time	t4	t1	t5	t5	t3	t3	t4	t4	t2	t1	t4	t2	t16	t16
Mean	144.88	146.18	149.70	149.70	166.88	167.14	171.14	171.14	171.77	179.10	180.85	182.83	184.84	187.35
Homogeneity	■	■	■	■	■	■	■	■	■	■	■	■	■	■
	■	■	■	■	■	■	■	■	■	■	■	■	■	■
	■	■	■	■	■	■	■	■	■	■	■	■	■	■
	■	■	■	■	■	■	■	■	■	■	■	■	■	■
										■	■	■	■	■

NPs	GO	GO	GO	GO	Ag	GO	GO	GO	Ag	GO	Ag	GO	Ag	Ag
Conc.	C1	C3	C3	C0	C0	C2	C3	C3	C3	C3	C2	C3	C3	C1
Time	t2	t21	t5	t21	t21	t5	t2	t3	t3	t16	t4	t1	t5	t3
Mean	188.17	190.12	191.95	192.30	192.30	192.55	196.05	198.42	202.13	209.19	222.31	222.50	222.83	223.88
Homogeneity	■	■	■	■	■	■	■	■	■	■	■	■	■	■
	■	■	■	■	■	■	■	■	■	■	■	■	■	■
	■	■	■	■	■	■	■	■	■	■	■	■	■	■
	■	■	■	■	■	■	■	■	■	■	■	■	■	■
	■	■	■	■	■	■	■	■	■	■	■	■	■	■
							■	■	■	■	■	■	■	■
										■	■	■	■	■
											■	■	■	■

NPs	Ag	Ag	GO	GO	Ag	Ag	Ag	Ag	Ag	Ag	Ag	Ag	Ag	GO
Conc.	C1	C3	C2	C2	C1	C2	C1	C2	C3	C1	C1	C2	C3	C1
Time	t2	t4	t3	t21	t4	t5	t21	t16	t16	t5	t16	t21	t21	t21
Mean	229.84	231.26	235.57	239.58	240.58	262.10	284.70	285.28	320.82	336.91	348.11	353.89	356.54	374.10
Homogeneity	■	■	■											
	■	■	■	■	■									
	■	■	■	■	■	■								
	■	■	■	■	■	■	■							
	■	■	■	■	■	■	■	■	■					
	■	■	■	■	■	■	■	■	■	■				
	■	■	■	■	■	■	■	■	■	■	■			
	■	■	■	■	■	■	■	■	■	■	■	■	■	
		■	■	■	■	■	■	■	■	■	■	■	■	■

Table S7. β -Glucosidase - Results of the Tukey post-hoc test performed after multivariate repeated measures Analysis of Variance (Homogeneous groups are marked with gray boxes).

Multivariate repeated measures Analysis of Variance – Tukey test (β-Glucosidase)

NPs	Ag	Ag	Ag	Ag	Ag	Ag	GO	GO	Ag	Ag	Ag	GO	Ag	GO	Ag	Ag	GO	Ag	Ag	GO	Ag	GO	Ag	GO	Ag	Ag	GO	GO	Ag	GO	Ag	Ag	GO	Ag	GO	GO	GO	Ag	GO	GO	GO	GO	Ag	GO	GO	Ag	GO	Ag	GO	Ag	GO	Ag	GO	GO	GO	GO
Conc.	C2	C2	C2	C2	C2	C0	C0	C0	C0	C2	C3	C1	C0	C0	C1	C2	C3	C1	C3	C1	C1	C1	C0	C0	C1	C0	C0	C3	C3	C0	C0	C1	C1	C3	C3	C1	C2	C3	C1	C1	C3	C0	C0	C2	C2	C1	C3	C3	C2	C1	C2	C3	C3	C3	C2	C2
Time	t5	t16	t2	t3	t4	t16	t16	t21	t21	t1	t5	t1	t3	t3	t2	t21	t4	t4	t4	t5	t16	t16	t5	t5	t1	t4	t4	t3	t16	t1	t1	t3	t2	t2	t21	t3	t4	t3	t21	t4	t5	t2	t2	t21	t1	t21	t1	t1	t5	t5	t16	t21	t2	t16	t3	t2
Mean	11.20	16.50	17.93	18.34	18.59	27.45	27.45	27.96	27.96	28.42	28.46	29.87	30.42	30.42	31.32	31.54	31.88	32.06	32.96	33.80	34.24	35.72	37.12	37.12	37.21	37.51	37.51	38.70	38.77	38.81	38.81	39.15	40.06	44.51	44.54	44.65	45.19	45.76	46.39	49.78	50.50	51.51	51.51	52.46	52.63	53.54	54.64	56.65	61.10	62.67	63.26	67.20	67.71	69.74	80.55	86.96
Homogeneity	■	■	■	■	■	■	■	■	■	■	■	■	■	■	■	■	■	■	■	■	■	■	■	■	■	■	■	■	■	■	■	■	■	■	■	■	■	■																		
		■	■	■	■	■	■	■	■	■	■	■	■	■	■	■	■	■	■	■	■	■	■	■	■	■	■	■	■	■	■	■	■	■	■	■	■	■	■	■	■	■	■	■	■											
			■	■	■	■	■	■	■	■	■	■	■	■	■	■	■	■	■	■	■	■	■	■	■	■	■	■	■	■	■	■	■	■	■	■	■	■	■	■	■			■	■											
					■	■	■	■	■	■	■	■	■	■	■	■	■	■	■	■	■	■	■	■	■	■	■	■	■	■	■	■	■	■	■	■	■	■	■	■	■	■	■	■	■											
						■	■	■	■	■	■	■	■	■	■	■	■	■	■	■	■	■	■	■	■	■	■	■	■	■	■	■	■	■	■	■	■	■	■	■	■	■	■	■	■	■	■	■	■		■					
								■	■	■	■	■	■	■	■	■	■	■	■	■	■	■	■	■	■	■	■	■	■	■	■	■	■	■	■	■	■	■	■	■	■	■	■	■	■	■	■	■	■	■	■					
																			■	■	■	■	■	■	■	■	■	■	■	■	■	■	■	■	■	■	■	■	■	■	■	■	■	■	■	■	■	■	■	■	■	■	■			
																						■	■	■	■	■	■	■	■	■	■	■	■	■	■	■	■	■	■	■	■	■	■	■	■	■	■	■	■	■	■	■	■	■		
																																		■	■	■	■	■	■	■	■	■	■	■	■	■	■	■	■	■	■	■	■	■	■	
																																								■	■	■	■	■	■	■	■	■	■	■	■	■	■	■	■	■

Table S8. β -Galactosidase - Results of the Tukey post-hoc test performed after multivariate repeated measures Analysis of Variance (Homogeneous groups are marked with gray boxes).

Multivariate repeated measures Analysis of Variance – Tukey test (β-Galactosidase)

NPs	Ag	GO	GO	Ag	Ag	Ag	Ag	GO	Ag	GO	Ag	Ag	Ag	Ag	GO	Ag	Ag	Ag	Ag	GO	Ag	GO	Ag	Ag	GO	GO	Ag	GO	Ag	GO	GO	GO	Ag	GO	GO	GO	GO	Ag	Ag	Ag	GO	Ag	GO	Ag	GO	GO	GO	GO	GO	Ag	GO	Ag	Ag	GO	GO	GO
Conc.	C3	C3	C0	C0	C3	C2	C0	C0	C3	C0	C0	C3	C3	C3	C2	C2	C1	C3	C1	C0	C0	C2	C2	C2	C2	C1	C0	C0	C0	C0	C2	C1	C1	C2	C1	C2	C1	C2	C1	C2	C3	C1	C2	C2	C1	C3	C1	C1	C3	C0	C0	C1	C1	C3	C3	C3
Time	t2	t4	t3	t3	t5	t2	t2	t2	t21	t16	t16	t16	t3	t1	t1	t3	t3	t4	t2	t4	t4	t4	t16	t4	t2	t4	t1	t1	t21	t21	t3	t16	t1	t5	t2	t16	t3	t1	t16	t5	t5	t4	t21	t21	t5	t3	t1	t21	t1	t5	t5	t21	t5	t2	t21	t16
Mean	1.747	1.789	1.859	1.859	1.966	2.026	2.081	2.081	2.167	2.196	2.196	2.319	2.322	2.343	2.370	2.392	2.410	2.417	2.464	2.491	2.491	2.515	2.517	2.599	2.611	2.646	2.660	2.660	2.802	2.802	2.836	2.896	2.932	2.999	3.074	3.110	3.175	3.189	3.264	3.312	3.360	3.405	3.424	3.584	3.596	3.600	3.655	3.719	3.790	3.809	3.809	3.809	4.282	4.508	4.525	5.406
Homogeneity																																																								

Table S9. Protease - Results of the Tukey post-hoc test performed after multivariate repeated measures Analysis of Variance (Homogeneous groups are marked with gray boxes).

Multivariate repeated measures Analysis of Variance – Tukey test (Protease)

NPs	Conc.	Time	Mean
GO	C2	t21	0.1214
Ag	C2	t16	0.1364
Ag	C3	t16	0.2821
Ag	C1	t21	0.2884
GO	C3	t21	0.3463
Ag	C1	t16	0.4150
GO	C1	t16	0.4171
Ag	C2	t21	0.4227
GO	C2	t16	0.4354
Ag	C1	t5	0.4598
Ag	C3	t21	0.4707
GO	C3	t16	0.4714
GO	C1	t21	0.4728
Ag	C2	t2	0.4778
GO	C1	t5	0.4821
GO	C0	t16	0.4839
Ag	C0	t16	0.4839
Ag	C2	t1	0.4881
Ag	C2	t3	0.4904
Ag	C0	t21	0.5079
GO	C0	t21	0.5079
GO	C1	t4	0.5193
Ag	C2	t4	0.5195
Ag	C1	t4	0.5203
GO	C3	t5	0.5295
Ag	C3	t5	0.5305
Ag	C1	t2	0.5348
Ag	C3	t1	0.5362
Ag	C1	t3	0.5452
Ag	C3	t3	0.5460
Ag	C2	t5	0.5467
GO	C3	t3	0.5584
GO	C2	t5	0.5647
Ag	C1	t1	0.5727
GO	C2	t3	0.5748
GO	C3	t4	0.5782
Ag	C3	t2	0.5968
GO	C0	t3	0.5995
Ag	C0	t3	0.5995
GO	C1	t2	0.6138
Ag	C0	t1	0.6180
GO	C0	t1	0.6180
GO	C2	t2	0.6188
GO	C3	t1	0.6219
GO	C3	t2	0.6221
Ag	C0	t2	0.6236
GO	C0	t2	0.6236
Ag	C3	t4	0.6263
Ag	C0	t5	0.6316
GO	C0	t5	0.6316
Ag	C0	t4	0.6398
GO	C0	t4	0.6398
GO	C1	t3	0.6430
GO	C2	t1	0.6483
GO	C2	t4	0.6536
GO	C1	t1	0.6571

Homogeneity

Table S10. Lipase - Results of the Tukey post-hoc test performed after multivariate repeated measures Analysis of Variance (Homogeneous groups are marked with gray boxes).

Multivariate repeated measures Analysis of Variance – Tukey test (Lipase)

NPs	Conc.	Time	Mean	Homogeneity 1	2	3	4	5	6	7	8	9	10	11
GO	C1	t3	0.001804	X	X	X	X	X	X					
GO	C1	t4	0.002120	X	X	X	X	X	X					
GO	C0	t2	0.003166	X										
Ag	C0	t2	0.003166	X										
Ag	C0	t21	0.003214	X										
GO	C0	t21	0.003214	X										
GO	C0	t5	0.003499	X										
Ag	C0	t5	0.003499	X										
GO	C0	t4	0.003524	X										
Ag	C0	t4	0.003524	X										
Ag	C0	t3	0.004057	X										
GO	C0	t3	0.004057	X										
Ag	C0	t16	0.004158	X										
GO	C0	t16	0.004158	X										
GO	C1	t5	0.004606	X	X	X	X	X	X					
GO	C0	t1	0.005511	X	X									
Ag	C0	t1	0.005511	X	X									
GO	C1	t2	0.005637	X	X	X	X	X	X	X	X			
Ag	C1	t1	0.006016	X										
GO	C1	t16	0.006608	X	X	X	X	X	X	X	X			
GO	C3	t16	0.007034	X	X	X								
GO	C3	t4	0.009694	X	X	X	X	X						
Ag	C1	t3	0.010267	X	X	X		X						
GO	C3	t2	0.010677	X	X	X	X	X						
Ag	C3	t2	0.012571	X	X	X	X	X	X					
GO	C2	t1	0.013818	X	X	X	X	X	X					
GO	C2	t4	0.013819	X	X	X	X	X	X					
GO	C2	t2	0.014206	X	X	X	X	X	X					
GO	C3	t3	0.014872	X	X	X	X	X	X	X	X			
GO	C2	t5	0.015247	X	X	X	X	X	X		X			
GO	C2	t16	0.017766	X	X	X	X	X	X	X	X			
GO	C1	t21	0.018610	X	X	X	X	X	X	X	X	X		
GO	C2	t3	0.019214	X	X	X	X	X	X	X	X	X		
GO	C1	t1	0.019877	X	X	X	X	X	X	X	X	X		
Ag	C2	t3	0.020023	X	X	X	X	X	X	X	X	X		
GO	C3	t21	0.020129	X	X	X	X	X	X	X	X	X		
Ag	C3	t5	0.020791	X	X	X	X	X	X	X	X	X		
Ag	C2	t4	0.021131	X	X	X	X	X	X	X	X	X		
GO	C3	t5	0.021213	X	X	X	X	X	X	X	X	X		
Ag	C1	t2	0.021400	X	X	X	X	X	X	X	X	X		
Ag	C1	t21	0.021630	X	X	X	X	X	X	X	X	X		
Ag	C1	t4	0.022200	X	X	X	X	X	X	X	X	X		
Ag	C2	t5	0.022479	X	X	X	X	X	X	X	X	X		
Ag	C2	t2	0.023869	X	X	X	X	X	X	X	X	X		
Ag	C1	t16	0.026820		X	X	X	X	X	X	X	X		
Ag	C1	t5	0.028253			X	X	X	X	X	X	X		
Ag	C3	t1	0.029130			X	X	X	X	X	X	X		
GO	C2	t21	0.030379				X		X	X	X	X		
GO	C3	t1	0.030491				X	X	X	X	X	X		
Ag	C3	t3	0.033224						X	X	X	X		
Ag	C2	t1	0.036919							X		X	X	
Ag	C3	t4	0.036942							X	X	X	X	
Ag	C2	t21	0.038518									X	X	
Ag	C2	t16	0.055957										X	
Ag	C3	t21	0.056860										X	
Ag	C3	t16	0.088583											X
